# ‘Imagined guilt’ *vs* ‘recollected guilt’: implications for fMRI

**DOI:** 10.1093/scan/nsw001

**Published:** 2016-01-08

**Authors:** Neil Mclatchie, Roger Giner-Sorolla, Stuart W. G. Derbyshire

**Affiliations:** ^1^ School of Psychology, Lancaster University, Lancester, UK,; ^2^ School of Psychology, University of Kent, Kent, UK, and; ^3^ Department of Psychology and A*STAR-NUS Clinical Imaging Research Centre, National University of Singapore, Singapore

**Keywords:** guilt, memories, hypothetical scenarios

## Abstract

Guilt is thought to maintain social harmony by motivating reparation. This study compared two methodologies commonly used to identify the neural correlates of guilt. The first, imagined guilt, requires participants to read hypothetical scenarios and then imagine themselves as the protagonist. The second, recollected guilt, requires participants to reflect on times they personally experienced guilt. In the fMRI scanner, participants were presented with guilt/neutral memories and guilt/neutral hypothetical scenarios. Contrasts confirmed a priori predictions that guilt memories, relative to guilt scenarios, were associated with significantly greater activity in regions associated with affect [anterior cingulate cortex (ACC), Caudate, Insula, orbital frontal cortex (OFC)] and social cognition [temporal pole (TP), precuneus). Similarly, results indicated that guilt memories, relative to neutral memories, were also associated with greater activity in affective (ACC, amygdala, Insula, OFC) and social cognition (mPFC, TP, precuneus, temporo-parietal junction) regions. There were no significant differences between guilt hypothetical scenarios and neutral hypothetical scenarios in either affective or social cognition regions. The importance of distinguishing between different guilt inductions inside the scanner is discussed. We offer explanations of our results and discuss ideas for future research.

## Introduction

A considerable body of research has demonstrated that guilt is elicited following a transgression against another individual or group and will influence subsequent moral decisions and moral behaviour (Trivers, 1971; Haidt, 2003). Guilt can, motivate individuals to act in a reparative (Tangney and Dearing, 2002; Ketelaar and Au, 2003; Nelissen, 2011) or generally prosocial manner (Regan *et al*., 1972).

The motivational component of guilt is generally considered to be part of the complex emotional experience that constitutes guilt (Baumeister *et al.*, 2007; Izard, 2007). Guilt can be understood as an ‘emotion schema’ (Izard, 2007), involving interactions of self-directed negative affect with self/other distinction, agency, counterfactual thinking, regret and future planning. It is this interaction of emotion with cognition that is believed to deliver the powerful motivation to act.

Findings from neuroimaging support the understanding of guilt as involving a complex interaction of affect and cognition (see Kédia *et al.*, 2008 for review). Multiple studies have shown that feelings of guilt activate affect-related regions including the anterior cingulate cortex (ACC; Shin *et al.,* 2000; Kédia *et al*., 2008), the orbital frontal cortex (OFC; Moll and de Oliveira-Souza, 2007; Zahn *et al.*, 2009; Morey *et al*., 2012), the insula (Shin *et al*., 2000; Wagner *et al.*, 2011; Michl *et al*., 2014), the amygdala (Berthoz *et al*., 2006; Kédia *et al*., 2008) and the basal ganglia (Kédia *et al*., 2008).

One or more of these regions have been activated during experiments involving: the perception of emotional stimuli, including facial expressions (e.g. amygdala, Hariri *et al*., 2002; Hare *et al*., 2005) and speech (e.g. basal ganglia, Pell and Leonard, 2003; Paulmann *et al.*, 2008); changes in, and awareness of, physiological arousal (e.g. insula, Critchley *et al.*, 2004; amygdala, Gläscher and Adolphs, 2003); motivation, including updating motivational states (e.g. ACC, Wager and Feldman-Barrett, 2004) and connecting motivational goals with visual information (e.g. basal ganglia, Kawagoe *et al*., 1998); reinforcing behaviours (e.g. the OFC, Bechara *et al*., 2000; O’Doherty *et al*., 2001; ACC, Bush *et al*., 2002; Etkin *et al*., 2011) and memory encoding (e.g. amygdala, Canli *et al*., 2000; Buchanan, 2007) and subsequent retrieval of emotional events (e.g. OFC, amygdala, (Maratos *et al*., 2001).

Social cognition networks include the medial prefrontal cortex (mPFC; Takahashi *et al*., 2004; Finger *et al*., 2006; Kédia *et al*., 2008; Basile *et al*., 2011; Morey *et al*., 2012), dorsolateral prefrontal cortex (Stone *et al*., 1998), temporo-parietal junction (TPJ; Finger *et al*., 2006; Kédia *et al*., 2008), the temporal poles (TPs; Shin *et al*., 2000; Finger *et al*., 2006; Wagner *et al*., 2011) and the precuneus (Takahashi *et al*., 2004; Moll and de Oliveira-Souza, 2007; Kédia *et al*., 2008). Social cognition networks are broadly associated with processing social information, including: perception (e.g. mPFC, Harris and Fiske, 2007; TPJ, Pelphrey and Carter, 2008), attention (e.g. TPJ, Nummenmaa and Calder, 2009) and storage and retrieval (e.g. precuneus, Cavanna and Trimble, 2006; TP, Olson *et al*., 2012). Social cognition networks, such as the TP (Olson *et al*., 2007), the mPFC (Gallagher *et al*., 2000; Dodell-Feder *et al*., 2011) and the TPJ (Saxe, 2010), are activated during experiments where participants imagined what others might be thinking or feeling or where participants generally take the perspective of another. Specific regions in social cognition networks activated during episodes of guilt include the mPFC (Takahashi *et al*., 2004; Finger *et al*., 2006; Kédia *et al*., 2008; Basile *et al*., 2011; Morey *et al*., 2012), the temporal poles (Shin *et al*., 2000; Finger *et al*., 2006; Wagner *et al*., 2011), the precuneus (Takahashi *et al*., 2004; Moll and de Oliveira-Souza, 2007; Kédia *et al*., 2008) and the TPJ (Finger *et al*., 2006; Kédia *et al*., 2008).

Thus, the neuroimaging literature is often interpreted as demonstrating guilt to involve negative affect combined with other-directed cognitions, or other variation of the ‘emotion schema’ described by Izard (2007). This interpretation, however, cannot be fully justified because the limitations of neuroimaging have so far prevented direct assessment of guilt as an emotion schema.

A major limitation of neuroimaging research is the necessity for participants to remain stationary. Even small movements or rotations of the head render the images unusable. Consequently, the elaborate set-ups and manipulation tasks commonly employed by social psychologists to induce feelings of guilt (Regan *et al*., 1972; Nelissen and Zeelenberg, 2009) are not transferable for use with neuroimaging. To accommodate the limitations of neuroimaging, two methods of inducing guilt within the scanner have primarily been used: the ‘memory recollection task’ and the ‘hypothetical scenario task’. Supplementary Table S1 summarises the different results when using these two methods to examine the neural correlates of guilt.

Studies involving the memory recollection task typically ask participants to recall a time that they transgressed against another individual. The memory recollection task is commonly used in behavioural experiments to induce feelings of guilt (Ketelaar and Au, 2003; Zhong and Liljenquist, 2006; Bastian *et al*., 2011) but has been used much less frequently inside the scanner (e.g. see Shin *et al*., 2000; Wagner *et al*., 2011). Participants recalled personal events that induced feelings of guilt and these events were presented to the participants during scanning to capture the neural correlates of guilty feelings. These two studies demonstrated common activation in the insula and TP cortices.

Studies involving the hypothetical scenario task typically ask participants to imagine they are the main protagonist in a hypothetical scenario describing a guilt-inducing event. Unlike recollection, hypothetical scenarios are not directly about the participant’s personal behaviour, and there is less evidence that hypothetical scenarios induce feelings of guilt. A central component of guilt is the awareness of one’s own responsibility for having committed a transgression against another person or group. When asked to consider hypothetical scenarios, the participant is not truly responsible for any transgression or harm. Consequently, the response to a hypothetical scenario is likely to involve anticipatory thoughts about guilt or the concept of guilt (‘guilt thoughts’) rather than a feeling of guilt (‘guilt feelings’), which might be expected to generate considerably different neural activation. Only one study of guilt using hypothetical scenarios demonstrated activation of the insular cortex (Michl *et al*., 2014) and only one activated the TP cortex (Finger *et al*., 2006). Michl *et al.* (2014) used hypothetical scenarios to elicit feelings of guilt and shame but noted as a limitation of their study that they could not guarantee the ‘success of imagination and generation of moral feelings’ (p. 155).

Importantly, guilt thoughts lack the painful, self-directed negative affect that is central to guilt feelings. This difference may also impact their motivational consequences. Although guilt feelings predict reparative or prosocial behaviours (Ketelaar and Au, 2003; Nelissen, 2012), guilt feelings can also motivate dysfunctional behaviour including self-punishment (Bastian *et al*., 2011) and anti-social behaviours (de Hooge *et al*., 2011). The self-directed negative affect of guilt feelings may motivate negative-state relief that conflicts with the motivation for prosocial behaviours (Miller, 2010 for a review).

In contrast, guilt thoughts have been shown consistently to motivate prosocial behaviours. Anticipating guilt has been associated with increased charity donations (Lindsey, 2005) and a decreased likelihood of cheating in exams (Malinowski and Smith, 1985). Other studies have shown that subtly making the concept of guilt accessible can promote reparative and prosocial behaviours (Zemack-Rugar *et al*., 2007; Giner-Sorolla, 2001). The cognitive reflection associated with guilt thoughts may motivate actions to prevent guilt in the future and thus motivate prosocial behaviours (Baumeister *et al*., 2007).

It seems likely, therefore, that neuroimaging studies of guilt feelings *vs* guilt thoughts will produce different activations as suggested by the summary in Supplementary Table S1. To date, no study has directly tested for differences between guilt feelings as induced by memory recollection and guilt thoughts as induced by hypothetical scenarios. Instead, studies have focused on different components of guilt, e.g. deontological *vs* altruistic (Basile *et al*., 2011), presence *vs* absence of an audience (Finger *et al*., 2006), target of agency (Kédia *et al*., 2008) and whether the outcomes are self- or other-oriented (Morey *et al*., 2012).

A direct comparison of memory recollection and hypothetical scenarios will be obviously more definitive than the comparison in Supplementary Table S1 because a direct comparison will eliminate confounds such as differences in sample sizes and thresholding across studies. The average sample size of the eight studies in Table 1 equals 15.1, but only three of the studies in Table 1 had a sample size greater than this. There are also considerable differences in methods of analysis. Seven studies employed whole brain analysis with thresholds ranging from *P* < 0.05 (Michl *et al*., 2004; Finger *et al*., 2006; Zahn *et al*., 2009; Morey *et al*., 2012) to *P* < 0.005 (Takahashi *et al*., 2004) to *P* < 0.001 (Wagner *et al*., 2011). Two studies used small volume corrections (SVCs) with a corrected *P*-value threshold < 0.001 (Shin *et al*., 2000; Kédia *et al*., 2008) and one study combined whole brain analysis (*P* < 0.001) and small volume analysis (P < 0.05) (Berthoz *et al*., 2006).

**Table 1.** The name, hemisphere, and z-score of regions associated with increased activity following guilt memories/neutral memories contrasts

	Guilt memory reflect *vs* neutral memory reflect			
Figure label	Brain areas (x,y,z)	Hemisphere	Z-score	Voxels
1	OFC*^a^**^ ^*(36, 24, −16)(BA47)	R	3.85	21
2	ACC*^a^**^ ^*(−6, 50, 4)(BA32)	L	4.44	157
3	Amygdala*^a^^ ^*(26, −4, −14)	R	3.92	6
4	Insular*^a^**^ ^*(36, 20, −14) (BA47)	R	4.40	87
5	Medial prefrontal cortex*^a^**^ ^*(−4, 56, 28)(BA9)*^ ^*(8, 54, 24)(BA9)	L*^ ^*R	5.24*^ ^*4.79	1145*^ ^*1145
6	TPs*^a^**^ ^*(−28, 16, 18)(BA13)*^ ^*(50, 10, −28)(BA21)	L*^ ^*R	4.00*^ ^*4.67	130*^ ^*177
7	Precuneus*^a^**^ ^*(−6, −52, 30)(BA31)	L	4.22	504
8	Temporo-Parietal Junction*^a^**^ ^*(−50, −60, 28)	L	4.17	104

^a^Indicates ROI, Peak-level threshold *p*_corr_<0.05, >23 contiguous voxels. Coordinates (x, y, z) are in MNI space (Montreal Neurological institute).

Given these differences, it is probably not surprising that Supplementary Table S1 does not indicate any single structure as significantly active across all studies. Nevertheless, it is notable that the two studies that used memory recollection reported significant activity in both affect-related and social cognition structures. This is precisely what would be predicted if guilt feelings induced by memory recollection involve structures associated with affect and social cognition. Of the studies that employed the hypothetical scenario, two studies support the hypothesis that guilt thoughts should not result in increased activity of affect-related structures.

An additional issue is that studies comparing guilt with control scenarios have not taken care to ensure that control scenarios are equal in social content to guilt scenarios. This is an important confound. The times when the literature does show the activation of, in particular, social cognition structures could thus merely be a function of the incidental social background involved in imagining someone else, not a key component of guilt as opposed to neutral but equally social situations. We therefore thought it was important to eliminate this confound by using neutral social situations as our control group to compare with guilt.

Our study assessed the distinction between guilt thoughts and guilt feelings within the same design by drawing on recalled or anticipated guilt-evoking situations, using fMRI. In addition to the improvement of including a social thought control condition, we took care to ensure that anticipated guilt thoughts were not incidentally drawing on actual memories of guilt feelings, by having the anticipated situations be intentionally chosen as dissimilar to existing experiences.

## Methods

### Participants

Twenty-five right-handed students from the University of Birmingham (mean age = 25.7, four males) took part in the study in exchange for £28 compensation. All participants provided written consent and were fully debriefed at the conclusion of the experiment. No participant had a history of neurological, psychiatric or other chronic clinical disorder.

### Pre-scanning session

Participants were asked to provide a written description of 10 specific memories: 5 instances of a time that they had caused harm or distress to another person, and 5 instances of an emotionally neutral event. Participants typed their memories directly in to a Word document. They were asked to use 50–70 words for each description.

The memories were then serially presented to participants on a computer screen with their ten descriptions in a pseudo-random order (E-Prime). Participants rated the memories on a scale of how guilty each made them feel (0 not at all, 6 extremely guilty). Participants also rated the extent they felt that the behaviour in each memory had violated a moral or social code (1 - not at all, 5 - completely broke a social or moral code).

Participants were then presented with 28 hypothetical scenarios (see Supplemental Materials 2; Supplementary Data). The scenarios were carefully matched so that all scenarios described a social event involving the protagonist and at least one other. This ensured that differences in neural activity were not the product of differences in the social content of guilt and neutral hypothetical scenarios (Finger *et al*., 2006). Sixteen of the scenarios described a situation in which the self violates a moral or social value (A), and 12 described an emotionally neutral event (B). For example:

A. Getting on to a packed train, you decide to sit in the priority seats, even though they are supposed to be given to more needy people than you, and there are elderly people standing up. After a few stops, you hear a bump. An elderly lady has fallen over. You realise you should have given your seat to her.

B. In order to get to university, you walk to your nearest bus stop. On the way, you bump in to a class mate who is also going to the bus stop. You have a conversation about the planned lessons and she tells you that she is going to town in the evening. After the bus journey, you both go to your lesson.

These hypothetical scenarios were presented to participants one at a time in a pseudo-random order, and participants were asked to rate the extent that the hypothetical protagonists described in each scenario had broken a moral or social code using the same scale they had used for their own memories. Participants also rated the extent that they could identify with the hypothetical scenario’s main protagonist on a scale (1—not at all, 5—completely), and were told that being unable to identify with the protagonist could be the result of themselves not having committed the same act or one similar to it. Once completed, participants were debriefed, compensated and informed that they would be contacted regarding their participation in the fMRI stage of the experiment.

Each participant memory was matched with a hypothetical scenario according to their ratings of moral or social code violation. Exact matchings and ± 1 matchings were considered acceptable. When there was more than one hypothetical scenario that matched with the memory, the hypothetical scenario with which the participant could least identify with was selected. The decision to measure the extent that participants could identify with the hypothetical memory is novel to the current research. Atleast past research could have benefitted from including a measure of identification, it was essential for this study so as to control and minimize overlap between memories and scenarios.

Of the 25 participants who attended the first session, 20 were invited back for the scanning session based on successful memory-hypothetical pairings. That is, their ratings of the extent that their memories violated a social or moral code matched their ratings for the extent that a hypothetical scenario described a violation of a social or moral code. This resulted in five guilt memories and five hypothetical scenarios that participants believed involved an act that violated a moral or social code to the same extent, and five neutral memories and five neutral hypothetical scenarios, which participants did not feel described an event in which a moral or social code had been violated.

### fMRI paradigm

**Fig. 1.**
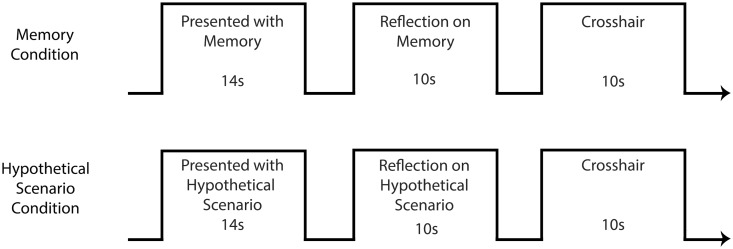
Demonstrates the functional imaging block design. Presentations lasted 14 s. Participants then reflected on the memory/scenario for 10s before being presented with a crosshair during which they were asked to clear their minds.

In the second session, participants were placed in an fMRI scanner. The study incorporated an epoch-based design with participants viewing and reflecting upon their memories for extended periods of time (>10 s). While in the scanner, memories (five guilty, five neutral) and hypothetical scenarios (five guilty, five neutral) were presented on a screen positioned directly in front of the participant and viewed in a mirror placed above the participant. The experiment was comprised of three runs. In a single run, participants would view all 20 presentations (10 memories, 10 hypotheticals) in a pseudo-randomised order. Each presentation consisted of three stages: ‘reading’ (14s) during which they were asked to read what was presented to them, ‘reflecting’ (10s) during which they were asked to reflect on the presentation that they had just read and then ‘control’ (10s), during which they were presented with a crosshair and instructed to empty their mind (see Figure 1). At the completion of the first two runs, participants were given a chance to get comfortable and relax before the next run started. Over the course of three runs, each memory was presented three times, for a total of 60 presentations. The experiment terminated following the third run. Participants were debriefed and received £25 compensation.

### Data acquisition

Functional data was acquired using a Philips 3 T Achieva system to acquire BOLD contrast weighted echoplanar images for the functional scans (repetition time TR = 3000 ms, echo time TE = 2000 ms, 48 sequentially acquired axial slices, 3-mm thick with a 3 × 3 mm in-plane resolution, FoV = 220 mm). High-resolution structural images were acquired using the T1TFE technique.

#### Preprocessing

Preprocessing and analysis of the data was conducted using SPM8 (Wellcome Department of Imaging Neuroscience, London). The preprocessing followed the same methodology outlined elsewhere (Derbyshire and Osborn, 2009). The first four fMRI volumes were discarded to allow for T1 equilibrium effects. Functional images were first corrected for differences in slice timing by resampling all slices with respect to the middle slice. Movement between scans was corrected for by spatially realigning each scan with the first, and these were then reoriented in to the standardised anatomical space provided by the MNI template. To complete the preprocessing, each image was smoothed in the X, Y and Z dimensions using a Gaussian filter of 8 mm full width at half maximum (FWHM).

#### fMRI statistical analysis

Standard neuroimaging methods based upon the general linear model were used for single participant analysis, which provided contrasts for group analysis at the second level. A box-car model convolved with a hemodynamic delay function was fitted to each voxel generating a statistical image corresponding to condition. Specifically, employing an epoch-based design, individual images were generated by subtracting BOLD activation of: (i) neutral memories from brain activation during reflection of guilt memories, (ii) neutral hypothetical scenarios from brain activation during reflection of guilt hypothetical scenarios (iii) guilt hypothetical scenarios from brain activation of guilt memories. Each of the subtractions was also reversed, subtracting BOLD activity during (iv) guilt memories from neutral memories, (v) guilt hypothetical scenarios from neutral hypothetical scenarios, (vi) and guilt memories from guilt hypothetical scenarios. These individual contrasts were then entered into a second level model to provide a group level significance map.

SVC was conducted across all contrasts for 10 pre-defined affective and social cognition regions. Specifically, a one-sample *t*-test was performed to assess group level bold activation using the contrast images generated at the individual level. Small volumes were predefined using the MRIcro atlas (www.mricro.com).

The Talairach and Tournoux (1988) atlas and the Talairach Daemon software (http://www.talairach.org/applet.html) were used to infer from the coordinates the region of activity. The coordinates were adjusted to allow for differences between the MNI and Talairach templates as outlined elsewhere (http://imaging.mrc-cbu.cam.ac.uk/imaging/ MniTalairach).

#### Thresholding

Whole brain images were thresholded at *p*_uncorr_ < 0.001, with an extent threshold of 23 contiguous voxels consistent with previous studies (Shin *et al*., 2000; Berthoz *et al.*, 2006; Kédia *et al*., 2008; Wagner *et al*., 2011). In addition, a series of mask images were created for each of the five affective regions (OFC, amygdala, insula, basal ganglia, ACC) and each of the four social cognition structures (mPFC, precuneus, TP, TPJ) identified a priori. The multiple comparisons problem was addressed through family-wise error (FWE) corrections for each mask separately (a SVC). fMRI activations were considered statistically significant if they exceeded a corrected threshold of *p*_fwe_ < 0.05. The coordinates of significant peak voxels and the size of the cluster were reported for each mask.

## Results

### Matching procedure

There was no significant difference between the extent that guilt memories (*M* = 3.47, SD* = *0.46) and guilt hypothetical scenarios (*M* = 3.54, SD = 0.42) were considered to have violated a moral or social code, *t*(19) = 1.02, *P* = 0.32. Similarly, there was no significant difference between the extent that neutral memories (*M* = 0.80, SD = 0.24) and neutral hypothetical scenarios (*M = *0.09, SD = 0.26) were considered to have violated a moral or social code, *t*(19) = 0.57, *P* = 0.58.

When more than one hypothetical scenario was considered to have violated a moral or social code to the same degree as a memory, the hypothetical scenario that participants could least identify with was chosen. Results showed that participants could identify with the neutral hypothetical scenarios (*M* = 2.59, SD = 1.79) significantly more than they could identify with the guilt hypothetical scenarios (*M* = 1.01, SD = 0.67), *t*(19) = 3.76, *P* = 0.001.

### Emotional ratings

Guilt feelings were significantly higher when participants reflected upon the guilt memories (*M* = 4.31) than when reflecting on the neutral memories (*M* = 0.13), *t*(18) = 27.07, *P* < 0.001. A mean rating of 4.31 corresponded to participants feeling ‘quite guilty’ while reflecting on their memories.

### fMRI data

The paradigm allowed for analysis of brain activity during the reading of each memory and hypothetical scenario, during reflection upon each memory and hypothetical scenario, and whilst observing the crosshair and being asked to empty their minds. Patterns of findings were similar for the reading and reflection periods of memories and hypothetical scenarios. The current methodology most closely resembles that of Wagner *et al*. (2011). In line with their research, here we present the analyses of the 10 s reflection period during which time participants reflected on the memory or hypothetical scenario they had just read.

### Guilt memories *vs* neutral memories

**Fig. 2.**
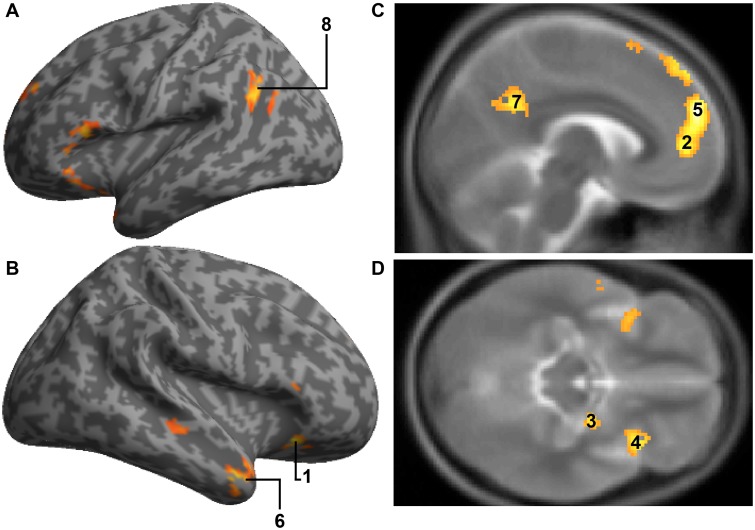
Results of the guilt memory/neutral memory contrast. effects are thresholded at *P* < 0.001, with a minimum cluster-size of 23 voxels. **(A)** Left hemispheric activity of the TPJ (8). **(B)** Right hemispheric activity of the OFC (1) and the Temporal Poles (6) **(C)** Sagittal view of hemispheric activity of ACC (2), mPFC (5) and precuneus (7). **(D)** Axial view of hemispheric activity of the amygdala (3) and insula (4).

When contrasted with neutral memories, guilt memories were associated with increased activity in both affective (OFC, ACC, Insula, Amygdala) and social cognition (mPFC, temporal poles, precuneus, TPJ) structures (see Figure 2, Table 1). Whole brain analysis revealed greater activation in the posterior cingulate and the inferior frontal cortices. There was no significant activity in the neutral memory condition when contrasted with the guilt memory condition.

### Guilt hypothetical *vs* neutral hypothetical

When contrasted with the neutral hypothetical scenarios, guilt hypothetical scenarios were not associated with significant increased activity in any structure of the brain. This was the case following whole brain and regional analysis. Furthermore, there was also no significant activity for the reverse contrasts; neutral hypothetical scenarios were not associated with increased activity when contrasted with hypothetical guilt scenarios.

### Guilt memories *vs* guilt hypothetical scenarios

**Fig. 3.**
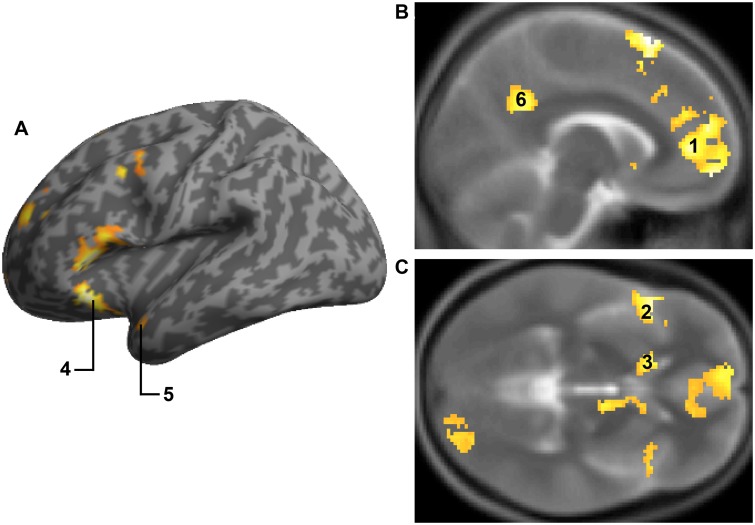
Results of the guilt memory/guilt hypothetical contrast. Effects are thresholded at *p* < 0.001, with a minimum cluster-size of 23 voxels. **(A)** Left hemisphere showing increased activity of the OFC (4) and the temporal pole (5). **(B)** Sagittal view showing increased activity of ACC (1) and the precuneus (6). **(C)** Axial view showing increased activity of the insula (2) and the caudate nucleus (3).

When neural activity during reflection on guilt memories was contrasted with neural activity during reflection on guilt hypotheticals, there was significantly more activity in both affective (OFC, ACC, caudate) and social cognition (mPFC, precuneus, superior temporal cortex) structures (Figure 3, Table 2). Additionally, other regions that were found to be significantly more active during the guilt memories compared with the guilt-laden hypothetical scenarios were the thalamus, the posterior cingulate, the primary motor cortex, and the inferior parietal cortex. No regions were significantly more active during the guilt-laden hypothetical scenarios when contrasted with the guilt memories.

**Table 2.** The name, hemisphere, and z-score of regions associated with increased activity following guilt memories/guilt hypothetical contrasts

	Guilt memory reflect *vs* guilt hypothetical reflect			
Figure label	Brain Areas (x,y,z)	Hemisphere	Z-score	Voxels
1	ACC*^a^**^ ^*(−8, 46, 6)*^ ^*(8, 30, 22)	L*^ ^*R	4.50*^ ^*3.90	896*^ ^*896
2	Insular*^a^**^ ^*(−44, 18, 2)*^ ^*(32, 18, −16)	L*^ ^*R	4.59*^ ^*4.60	212*^ ^*250
3	Basal Ganglia, caudate*^a^**^ ^*(−14, 18, −6)	L	4.68	94
4	OFC*^a^**^ ^*(−2, 66, −2)(BA10)*^ ^*(31, 20, 13)(BA13)	L*^ ^*R	4.01*^ ^*3.94	7*^ ^*7
5	Temporal Pole*^a^**^ ^*(−40, 18, −16)	L	4.66	310
6	Precuneus*^a^**^ ^*(−8, −50, 32)(BA31)	L	4.19	194

*^a^*Indicates ROI, peak-threshold *p*_corr_<0.05, >23 contiguous voxels. Coordinates (x, y, z) are in MNI space.

## Discussion

All participants provided personal accounts of recalled scenarios that involved neutral or guilt-related events (guilt memories). The experimenter generated hypothetical scenarios that also involved neutral or guilt-related events (guilt scenario). The guilt memories and guilt scenarios were successfully matched for the perceived extent to which they violated a moral or social code.

fMRI data revealed significant activation of affective (OFC, ACC, insula and amygdala) and social cognition (mPFC, temporal poles, precuneus and TPJ) structures after viewing guilt memories compared with neutral memories. There were no significant differences when comparing guilt scenarios and neutral scenarios. Direct comparison of activation following presentation of guilt memories with guilt scenarios confirmed greater activation of affective and social cognition structures after recalling guilt memories. These findings confirm the prediction that memories of personal events involving a moral violation will generate activity of both affect and social cognition structures.

In contrast to guilt memories, guilt scenarios did not activate either affect or social cognition structures significantly. Previous studies using hypothetical guilt scenarios have reported activation of social cognition structures (Takahashi *et al*., 2004; Berthoz *et al*., 2006; Finger *et al*., 2006; Kédia *et al*., 2008; Zahn *et al*., 2009; Basile *et al*., 2011; Morey *et al*., 2012). The absence of any significant activation in our study could be explained by the care we took to match both the guilt and neutral hypothetical scenarios so that each involved a social interaction. For example, one of our hypothetical guilt scenarios involved kicking a ball away from a group of children playing football while one of the neutral scenarios involved cutting a hedge for grandparents. Both scenarios involve social interaction and motor behaviour and so might, therefore, activate similar brain regions. In contrast, past studies have offered hypothetical scenarios with varying levels of social content. For example, Takahashi *et al*. (2004) presented participants with both solitary (‘I change in to pajamas at night’) and social (‘I betrayed my friend’) hypothetical scenarios. In this study, even though the guilt scenario involves a moral violation, the participant has no personal involvement or responsibility and therefore additional areas associated with guilty feelings are not activated.

The lack of activity in response to guilt scenarios does not support the suggestion that merely considering guilt-related events generates residual guilty feelings (Baumeister *et al.*, 2010). In this study, however, subjective feelings of guilt during scenarios were not recorded concurrently with imaging. It is possible that feelings of guilt were impacted by the guilt-scenarios but this impact was insufficient to generate significant neural activity. Although future studies may address this possibility by recording guilt feelings in response to all scenarios and directly correlating guilt feelings with neural activity, they would do so only under a sort of Heisenberg uncertainty paradox, as engaging in such a repetitive focus on guilt in self-report may increase its anticipation and accessibility beyond what is usual. Collecting guilt measures after the scan might have provided some information but the large number of scenarios led us to not attempt any *post-hoc* measures. Future studies might consider experimenting with *post-hoc* measures to at least provide some insight regarding subjective experience in the scanner.

In contrast to the lack of activation after viewing guilt scenarios, viewing guilt memories resulted in activation of both affect and social cognition structures. This finding suggests that guilt memories successfully resulted in generating both affective responses and recall of the events involving other people and is consistent with the understanding of guilt as an emotion schema. Merely thinking about guilt when presented with a hypothetical guilt scenario did not generate activity in affect related regions, suggesting that guilt thoughts do not involve an automatic affective component (Winkielman *et al.*, 2005). Moreover, in this study, reflecting on guilt scenarios also resulted in no additional social cognition activation, which suggests that the guilt thoughts were not markedly different than thoughts about other social situations.

Previous studies using guilt scenarios have demonstrated activation in affective or social cognition regions or both (see Supplementary Table S1). It is possible that these past studies inadvertently involved affect or cognitive triggers during the guilt scenarios that were effectively controlled in this study. For example, in this study, participants were presented with scenarios that they had previously rated very low in personal identification. This was to ensure that the distinction between memory and scenario was maintained. Ensuring that participants could not highly identify with the protagonist might also have avoided activating the affective and social cognition structures that were reported active in past studies. Indeed, it is possible that the low identification with the scenario led the participants to not engage strongly with the scenario and thus not generate any feelings of guilt at all. Low identity, however, does not mean low engagement and it is likely that the participants did engage with the scenarios. Participants were asked to imagine they were the protagonist and there is no reason to expect low identification to have prevented that imagined activity taking place; our participants did not report any difficulty in imagining what was happening and clearly were able to rate the scenarios for appropriateness, which indicates active engagement.

Furthermore, it is unlikely that past research generated greater identification with the protagonists for other hypothetical scenarios that have been used (e.g. attending dinner at a Japanese friend’s house, not liking the food and spitting it out on a plate, Berthoz *et al*., 2006; forgetting to validate a friend’s lottery ticket who had winning number, Kedia *et al*., 2008; or simply viewing an upset face; Basile *et al*., 2011). Moreover, given the similarity between the scenarios used in this study and previous research, it is unlikely that low engagement with the scenarios in this study fully explains the differences in activation. Nevertheless, future studies might include a mix of high and low identification scenarios to address this concern. Here we chose to restrict our study to personal memories and low identification scenarios to ensure that the activation during guilt hypotheticals was not due to overlap with personal memories and to provide maximal scan data to assess our central hypothesis.

A further limitation is that it is not possible to know from this study whether the guilt memories generated non-guilt related emotions such as frustration or despair that drove the affective activation. Similarly it is possible that the neutral scenarios also generated guilt-related emotions that negated any activation from the guilt scenarios and/or involved a complexity that was not adequately controlled by the guilt scenarios. Future studies might address these points by including additional controls and recording additional subjective measures.

Future research could also provide a rigorous quantitative meta-analysis of previous research. Several methodologies exist that researchers could employ to provide an overview of neural activity during episodes of guilt. These methods, such as activation likelihood estimation, work by pooling together the 3D coordinates of peak voxels reported as active across multiple studies and compare the observed number of peaks to a null hypothesis distribution (for a review of neuroimaging meta-analysis techniques, see Wager *et al*., 2007). Such a meta-analysis could provide better clarity than provided here in Supplementary Table S1 and address the issues pertaining to guilt raised by this study.

Past imaging research has not explicitly distinguished guilt thoughts and guilt feelings. The results of this study suggest researchers should be wary of drawing conclusions about emotions from studies based on hypothetical situations, as opposed to lived experience; and should also study social emotions using neutral scenarios with equal social content, in order to separate incidental confounds from true neural correlates of emotion.

## Funding

NM was supported by a joint Medical Research Council and Economic and Social Research Council Interdisciplinary Studentship, G0901456.

## Supplementary Material

Supplementary Data
