# Supplementary material for: ‘Imagined guilt’ vs ‘recollected guilt’: implications for fMRI
Source: Soc Cogn Affect Neurosci. 2016 Jan 8;11(5):703–11. doi: 10.1093/scan/nsw001 (PMC4847697; doi:10.1093/scan/nsw001)
Supplement: Supplementary Data [file nsw001_supplementary_data.zip › scan-15-285-File007.docx]

**Supplemental Material**

Supplemental Table 1 (S1)

*Functional imaging results from previous research showing increased activity of limbic structures and social cognition structures during guilt episodes.*

|  | |  |  |  | **Limbic Structures** | | | | | **Social Cognition Structures** | | | |
| --- | --- | --- | --- | --- | --- | --- | --- | --- | --- | --- | --- | --- | --- |
| **Study** | **N** | | **Threshold** | **Method of**  **Induction** | **ACC** | **OFC** | **Insular** | **Amygdala** | **Basal**  **Ganglia** | **mPFC** | **TP** | **Precuneus** | **TPJ** |
| Shin et al. (2000) | | 8 | Small Volume  *Z* >3.09,  *p*_uncor_< 0.001 | Memory | **↑*** |  | **↑*** |  |  |  | **↑*** |  |  |
| Wagner et al. (2011) | | 15 | Whole Brain  *p*_uncorr_< 0.001 | Memory |  |  | **↑** |  |  |  | **↑** |  |  |
| Takahashi et al. (2004) | | 19 | Whole Brain  *p*_uncorr_*<* 0.005 | Hypothetical |  |  |  |  |  | **↑** |  |  | **↑** |
| Berthoz et al. (2006) | | 12 | Whole Brain  *p*_uncorr_< 0.001,  Small Volume  *p*_uncorr_< 0.05 | Hypothetical | **↑** |  |  | **↑*** |  |  |  | **↑** | **↑** |
| Finger et al. (2006) | | 16 | Whole Brain  *p*_corr_< 0.05 | Hypothetical |  |  |  |  |  | **↑** | **↑** |  | **↑** |
| Kédia et al. (2008) | | 29 | Small Volume  *p*_uncorr_< 0.001 | Hypothetical | **↑*** |  |  | **↑*** | **↑*** | **↑*** |  | **↑*** | **↑*** |
| Zahn et al. (2009) | | 29 | Whole Brain  *p*_uncorr_< 0.05 | Hypothetical |  | **↑** |  |  | **↑** | **↑** |  |  |  |
| Basile et al. (2011) | | 22 | Whole Brain  *p*_corr_< 0.05 | Hypothetical | **↑** |  |  |  |  | **↑** |  |  |  |
| Morey et al. (2012) | | 16 | Whole Brain  *Z*>2.3,  *p*_corr_< 0.05 | Hypothetical |  | **↑** |  |  |  | **↑** |  |  |  |
| Michl et al. (2014) | | 14 | Whole Brain  *p*_fwe_< 0.05 | Hypothetical |  |  | **↑** |  |  |  |  |  |  |

* Small Volume Analysis

**Supplemental Material 2 (S2): Hypothetical Scenarios**

Guilt Scenarios

1. You are in a park with some friends and there are a group of children playing football a little distance away. After a few minutes their football rolls over to where you are located. When you go to kick it back to them, you accidently kick it up a tree. As a consequence, the children can no longer play football.

2. Getting on to a packed train, you decide to sit in the priority seats even though they are supposed to be given to more needy people than you, and there are elderly people standing up. After a few stops, you hear a bump. An elderly lady has fallen over. You realise you should have given your seat to her.

3. One evening, a smartly dressed man approaches you in the street. He has a badly bleeding nose and tells you he has just been mugged and had his wallet and phone stolen. He asks if he could have some change to phone someone. You tell him that you don’t have a phone or any change on you, knowing full well that you do.

4. Your partner accuses you of cheating. You lie to her, but they tell you they have read your text messages. This surprises you. Although you cheated, you still love them very much. You know they are very hurt and upset because of your infidelity. They ask you to leave immediately.

5. Your friend has a large DVD collection. You realise they probably would not notice if you took one without asking, especially as you know you can return it. When you get home you find that the contents of your bag has broken the case and had snapped your friends DVD.

6. Whilst out with your girlfriend, they give you their bag to look after whilst they go to the toilet. You really don’t want to have to walk home later, but don’t have any money for the bus. It occurs to you that you saw your girlfriend put her purse in her bag earlier. You decide to take some of the money before they come back.

7. One of your friends has been excited about cooking for you all week. They want you to try their food, and you are happy to go round and try it. When you put it in your mouth, you instantly dislike it and spit it out in front of them, screwing your face up as you do so. Your friend looks upset and you realise you have hurt their feelings.

8. Your young sister has recently achieved a 50 meter certificate at swimming. She is very proud and desperate to tell you about it. When she runs in to your room, you tell her you don’t care and to get out. She looks upset, and you realise you ruined her moment.

9. Working in a supermarket, a customer approaches you and asks you if there are any hay fever tablets in stock. You are feeling lazy and don’t want to go and look. Instead, you go out back and talk to a friend about sports. You then return to the customer and tell them the store has sold out.

10. You’re playing rugby against someone who is popular but you don’t like them. At one point during the game they have the ball and you tackle them violently. You find out that you have hurt their shoulder and they cannot continue to play. A few weeks later you see them out with your friends and see that they have their arm in a sling.

11. Whilst in class, a friend whispers to you something interesting about the topic. A moment later you put your hand up and say it. The teacher likes it and says it shows you have brains. You know you should say it was your friend’s idea, but you quite like the praise. You look at your friend and see they look upset.

12. You are on your way home after a night out with some friends. You realise one of your friends is shouting racist abuse at a group of people across the street. Although you are not racist, you decide to join in and say racist things at the other group, who seem upset but don’t retaliate. The next morning you wake up and realise how offensive your comments had been.

13. You visit your grandparents who have a box of chocolates on the table. You ask if you can have one, and they say that you can. However, when you reach in you purposefully hide three sweets in the palm of your hand but make it look like you only took one.

14. After doing a long distance run on the treadmill, you feel tired and desperate for water. You forgot your bottle, so drink from your friends. However, when your friend finishes shortly after you, you realise you have drank almost all of his water. He looks thirsty and a bit angry.

15. Your friend’s dog rushes in to the room and gets excited that you are there. You don’t like dogs, but you don’t want to tell your friend to get his dog away. When your friend goes to the toilet, the dog rushes over to your feet again, but this time you kick it. The dog is obviously hurt and leaves.

16. You and a friend are applying for the same promotion at work. One day after work you go to a pub with your boss. When your friend comes up in conversation, you subtly say disparaging things about him in order to make him look bad. A few days later you find out you got the promotion.

Neutral Scenarios

17. You go to your grandparent’s house and have dinner. They mention that it would be useful for them if you could cut the hedge in the afternoon, and after looking at the time, you say that you will. After dinner you find the hedge trimmer, go to the garden and trim the hedge.

18. You begin your morning by getting dressed before going down stairs and choosing which cereal you wish to eat. After making your choice, you get a bowl and pour the cereal in before adding milk. Just as you finish your cereal your housemate enters the kitchen and says good morning.

19. It is lunch time at work. You take out your lunchbox and eat lunch at your desk. You do this most days, because it allows you to leave earlier in the afternoon. Your boss normally asks you why you don’t take the hour off, but you just prefer to work the hours that you do.

20. You realise you don’t have much food left in the kitchen. You decide to go shopping. The trip to the supermarket isn’t particularly long. Because you know what you want to buy, you do your whole shop quite quickly. You go to the check out and the check out lady greets you as she starts scanning your items.

21. You have a new bike and have decided to ride it to university for the first time. Upon arriving, a fellow student in your class asks you how long you have had the new bike for. You tell her that you haven’t had it very long, and it is new.

22. In order to get to university, you walk to your nearest bus stop. On the way, you bump in to a class mate who is also going to the bus stop. You have a conversation about the planned lessons and she tells you that she is going to town in the evening. After the bus journey, you both go to your lesson.

23. Your friend asks you if you prefer salt and vinegar flavoured crisps or cheese and onion. You tell them you like both. She takes out a packet of crisps from her bag and gives them to you. She says she accidently packed two packets by accident. She has given you salt and vinegar.

24. You order a memory stick from online. It arrives a couple of days later. You decide to load your files on to it for convenience. The next time you go to university, you get the memory stick out of your bag and open up your work. After doing your work, you save the updated version to the memory stick.

25. Whilst in town, you decide to go browsing through some clothes shops. You don’t particularly need anything, but are willing to buy a bargain. The first shop you go to has some nice t-shirts reduced to quite a cheap price. You ask the lady in the store where the changing room is, and she tells you that they are downstairs on the ground floor.

26. You arrive at a cinema early. You are the first to get seats, and you and your friend are the only two people in the room. The lights are still on and there is some quiet background music playing. Your bag is by the side of your feet and you rest the popcorn on the arm rest.

27. After spending a day at university, you are about to head home. You walk to your car and get in. You place your bag on the passenger’s seat and put your seat belt on. You start the car, and the radio starts playing. You listen to an audiobook on your way home.

28. Whilst in the supermarket, you remember you have recently been short of Biros. You go to the aisle where there is lots of different types of stationary. The choice is between a pack of black pens or a pack of multicoloured pens. You choose the multicoloured pens because you want to write in green.
